# Transvenous removal of adherent hemodialysis catheters and venous ports – experience of a reference center

**DOI:** 10.34172/jcvtr.025.33544

**Published:** 2025-09-28

**Authors:** Janusz Gozdek, Dorota Nowosielecka, Wojciech Jacheć, Łukasz Tułecki, Andrzej Kutarski

**Affiliations:** ^1^Department of Cardiac Surgery, The Pope John Paul II Province Hospital, 22-400 Zamość, Poland; ^2^Faculty of Health Sciences, Academy of Zamość, Poland; ^3^2nd Department of Cardiology, Zabrze, Faculty of Medical Science in Zabrze, Medical University of Silesia in Katowice, Katowice, Poland; ^4^Department of Cardiology, University Hospital, 20-059 Lublin, Poland

**Keywords:** Transvenous catheter removal, Adherent femodialysis catheter, Adherent venous port

## Abstract

**Introduction:**

Removal of adherent intravascular catheters (hemodialysis catheters and venous ports) is still an unsolved clinical problem lying at the intersection of vascular surgery, anesthesiology, cardiac surgery and cardiology. Analysis of resistant removals of adherent catheters when simple traction was unsuccessful. Description of the technique and effectiveness of catheter removal using mechanical dilatation and dedicated tools.

**Methods:**

Retrospective review of a prospectively maintained computerized database at the reference center. One hundred eleven transvenous catheter extractions (TCE), including 71 hemodialysis catheters and 40 venous ports.

**Results:**

A procedure for removing adherent catheters using mechanical dilatation is described. All catheters were removed in their entirety, there was one major complication (embolization). It is difficult to identify predictors of the need for mechanical dilatation. The main indication for catheter removal is malfunction most frequently due to fibrous encapsulation at catheter tips or adherence of catheter tips to the cardiac structures. The second is catheter-related infection. Most dysfunctional and infected catheters are implanted with improper positioning of the catheter tip beyond the right atrium. Half of seemingly adherent catheters can be removed with simple traction, which is not predictable before the procedure. Moreover, 50% of catheters require dissection of fibrous tissue using additional specialized tools. The effects of mechanical dilatation are very good, if the procedure is performed with participation of operators experienced with transvenous lead extraction. TCE of adherent hemodialysis catheters and venous ports is a safe and effective procedure.

**Conclusion:**

The final result of mechanical dilatation is very good if the procedure is performed with collaboration of operators experienced with transvenous lead extraction. Transvenous removal of adherent hemodialysis catheters and venous ports is safe and effective.

## Introduction

 Increased life expectancy has increased the number of elderly people suffering life-long disability and multimorbidity that require various forms of medical intervention. Additionally, the advance of medical technologies and therapies, including cancer therapy or renal replacement therapy frequently requires placement of permanent venous access devices.^1^ The widespread use of hemodialysis catheters or vascular ports is associated multiple exchange or removal procedures. The most frequent indications for the removal of venous access devices are localized and systemic infections, catheter malfunction, dislodgement (entire devices or fragments), embolization, venous thrombosis and no longer needed catheters.^1-12^ These situations are associated with increased morbidity and mortality among patients.^2-12^

 Like endocardial leads hemodialysis catheters and venous ports become adherent to the vein or heart walls as a result of fibrin sheath formation. It develops over time and the main cause is irritation of the vascular endothelium by a foreign body (catheter, port) as well as administration of hypertonic fluids. Over time the connective tissue binding the catheter to the vein/heart wall becomes calcified. The adherent catheter/venous port cannot be removed using simple traction but only after mechanical dilation.^13-15^

 The increasing problem of removing adherent hemodialysis catheters or venous ports,^16^ prompted us to create a registry of patients undergoing transvenous extraction of such devices and then analyze indications for the procedure, risk factors as well as procedure effectiveness and safety.

 Indications for transvenous catheter extraction (TCE) can be divided into infectious and noninfectious. Infectious indications include localized and systemic infections together with catheter-related infective endocarditis.^6-12^ Noninfectious indications include catheter malfunction, dislodgement of an entire catheter or its fragment, systemic embolization (for instance catheter-related thrombosis, catheter-associated right atrial thrombus), venous thrombosis (including catheter-related superior vena cava syndrome), and no longer needed catheters.^3-4,7,11,17-22^

 This study focuses on removal of difficult catheters, more and more frequently encountered in clinical practice but rarely addressed in the literaturę.^23-25^

## Material and Methods

###  Study group

 The study group consisted of 111 patients undergoing TCE procedures at the same reference center between February 2015 and January 2025. Data were entered prospectively but analyzed retrospectively. Our computerized registry is open, data on new patients are entered on an ongoing basis. The TCE registry includes patients with adherent hemodialysis catheters and venous ports.

###  Transvenous catheter extraction (TCE)

**Figure 1:**
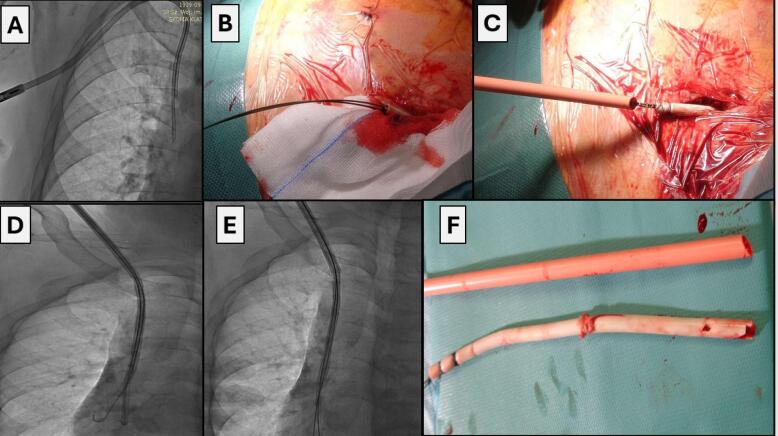
Removal of a hemodialysis catheter Removal of an 11-year-old infected hemodialysis catheter using mechanical dilatation with the largest polypropylene sheath dedicated for transvenous lead extraction. Stiffening of the removed catheter with standard guide wires (B-E) and ligating the catheter with guide wires to strain it during extraction (C,F)

**Figure 2:**
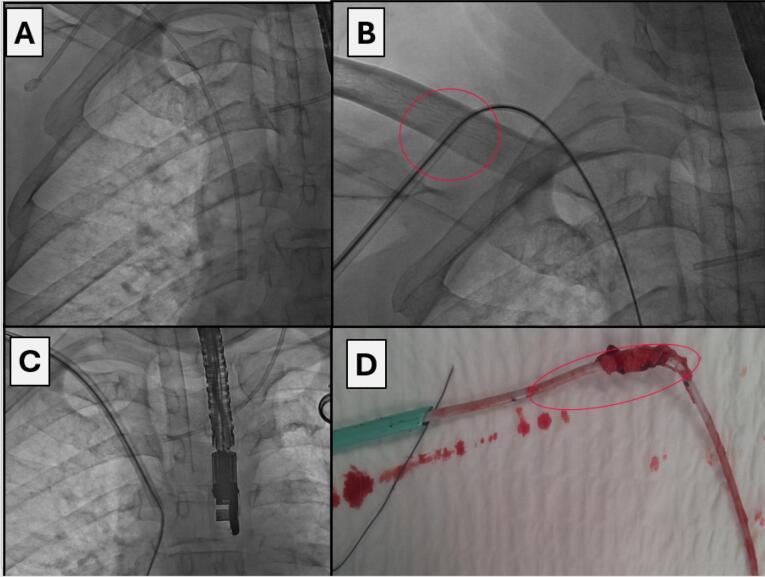
Removal of a venous port Removal of an 8-year-old inactive „nonremovable” catheter/venous port using mechanical dilatation with medium sized polypropylene sheaths dedicated for extraction of intracardiac leads. Fluoroscopy before the procedure (A). Stiffening of the catheter with a standard single guide wire (B,C). Panel D shows incised fibrous tissue which blocked port removal. The patient underwent also removal of another old venous port on the left side of the chest with simple traction due to termination of cancer therapy

**Figure 3:**
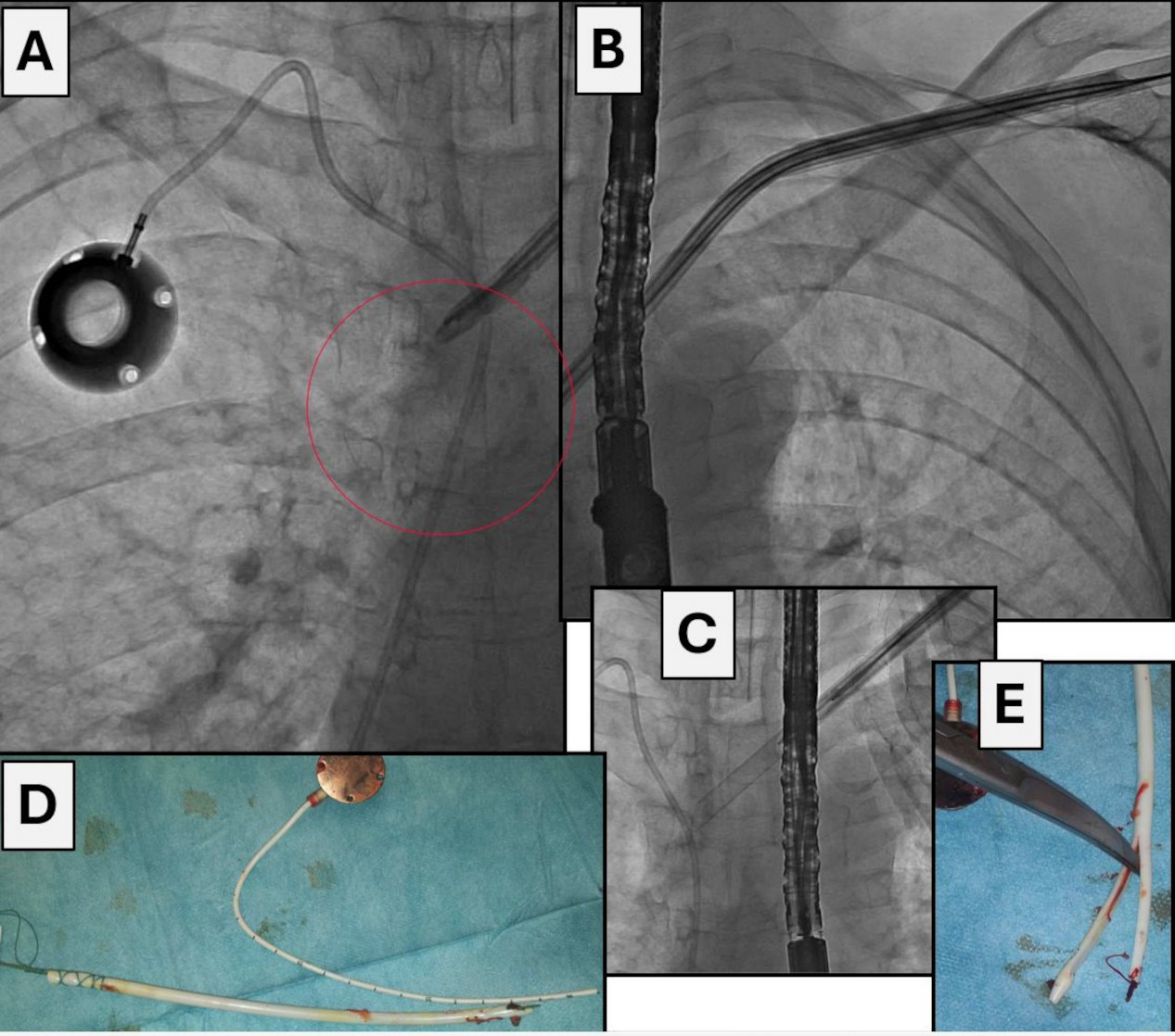
Removal of an adherent venous port and hemodialysis catheter Removal of a venous port – the right side of the chest (A) and removal of an infected hemodialysis catheter – the left side of the chest (B). Both devices in the common fibrous tissue block, removed. The catheters were separated using the largest polypropylene sheath dedicated for transvenous lead extraction (D). The tip of the hemodialysis catheter after removal (E)

 All procedures were performed in the hybrid operating room with vital signs monitoring. Depending on the type of expected technical difficulties the procedure was performed under regional or general anesthesia. In some cases phlebography was initially performed to determine venous patency if catheter/port exchange or additional venous stenting were planned. All procedures were performed under fluoroscopy control, and if indicated, under transesophageal echocardiographic guidance. The surgical field was prepared and draped as for a classic sternotomy. An innovative approach was catheter/port removal analogically to transvenous extraction of endocardial leads. It stemmed from the fact that like endocardial leads, hemodialysis catheters/venous ports interact with the surrounding structures. The technique of catheter extraction was determined by the extent of adherence to the vein or cardiac wall. First, after dissection of the catheter in the subcutaneous tissue (in case of a hemodialysis catheter it is necessary to dissect and cut off the felt cuff), we used direct traction, which appeared sufficient (during gentle pulling on the catheter it slowly came out from the fibrous tissue) [17-18]. Prior to removal one or two (depending on the number of channels) conventional angiographic guide wires were advanced to stiffen the catheter, which facilitated dissection and provided access to the vessel after catheter removal (Figure 1).^19^ The catheter was tied to the guide wire with a surgical ligature with single knots, alternately, about 10-15 times. In this way, when we pull on the guide wire, we also pull on the catheter (uniformly distributed forces prevent the ligature being cut off by the traction and the catheter sliding out (Figure 1, 2). In some cases the catheter is removed only with simple traction. After ligating the catheter to the guide wire, the operator slowly and uniformly pulls on the guide wire (with ligated catheter), the catheter becomes strained and elongated, and its diameter diminishes (at the same time the fibrous sheath causing catheter adherence to the vein and heart walls is loosened). This scheme of operation resulted in liberation of the catheter from the surrounding tissue and removal from the patient’s body. In case of failure, the next step was to use mechanical dilatation with polypropylene sheaths. Telescoping Byrd catheters (Byrd Dilator Sheaths, Cook Medical Inc., USA), are dedicated for transvenous lead extraction, here they were used to remove adherent hemodialysis catheters after gradual dissection from the vein and cardiac walls (Figure 1, 2, 3). If the catheter is placed close to the collarbone, it can adhere to the periosteum, in that case polypropylene sheaths are replaced with stainless steel sheaths (they can be used only when removing venous ports and parenteral nutrition catheters) (Figure 2). After dissection from the periosteum we continue dissection of the catheter with polypropylene sheaths (it is also safer). Sheath size (diameter) is adjusted to size (diameter) of hemodialysis catheters and venous ports.

 Sheaths are color-coded depending on their french sizes. Sheaths with (smallest) diameter 7Fr are blue, 8.5Fr yellow, 10Fr green, 11.5Fr white and 13Fr orange. When dissecting hemodialysis catheters we can use only orange sheaths because of their largest diameter, only these sheaths can be pulled over the hemodialysis catheter. Dissection of venous ports is begun with the smallest sheaths, and if there is no advancement, they are changed to larger ones. By using the sheaths, after catheter removal, the maintained vascular access allows for placement of the guide wire, followed by (depending on the indication and if there are no signs of infection) implantation of a new catheter.

 Ruptured hemodialysis catheters and venous ports or their fragments spontaneously dislodged into the circulation (pulmonary arteries) were removed with lasso catheters (loop snares) most frequently via the femoral access.^20-22^

 In the next step, after catheter removal, depending on the indications, a new catheter was implanted via the same venous access (angiographic guide wires left in place), alternative access (associated with puncture, creation of access to another central vein) or another procedure was performed i.e. dilatation and stenting of veins.

 Procedure safety was defined as the percentage of people with complications during or after the procedure compared with all patients undergoing the procedure. A major complication was defined as death or urgent sternotomy.

 Catheter malfunction was defined as too much resistance during blood withdrawal most frequently due to fibrous sheath formed at its tips or adherence to cardiac structures. This malfunction may also limit or stop a catheter function, which means that it can no longer be used. Other indications for catheter removal include localized infections diagnosed by the presenting symptoms such as edema, redness, excessive warmth, pus in the catheter vicinity (at the site of catheter passage through the skin or abscess in the subcutaneous tissue along the catheter) and culture results. Systemic infections were diagnosed if there was fever, elevated inflammatory markers and positive blood cultures. Catheter-related infective endocarditis was diagnosed according to the modified Duke’s criteria.^26^

###  Statistical analysis

Due to nonlinear distribution continuous data are presented as median and lower and upper quartiles and were analyzed using the Mann-Whitney U test. Categorical data are presented as number and percentage and were analyzed using the Chi^2^ test with Yates correction. A two-tailed *P* value < 0.05 was considered statistically significant. Statistical analysis was performed using STATISTICA 13.1 PL (TIBCO, Poland, Cracow).

## Results

Table 1 summarizes basic characteristics of the enrolled patients in the TCE registry. The mean age of patients undergoing transvenous extraction of hemodialysis catheters or venous ports was 59 years. Those undergoing removal of venous ports were mainly women (70.3%), whereas removal of hemodialysis catheters was more likely in men (57.14%). Hemodialysis catheters were most frequently implanted on the right side of the chest (61.26% vs 35.14%). The most common indication for catheter extraction was catheter malfunction (56.76%), infections (41.23%) and no longer needed catheters (10.81%). CIEDs (PM/ICD/CRT) were implanted in 17.11% of patients. It is noteworthy that half of hemodialysis catheters and more than a half of venous ports were implanted with improper positioning of their distal tips beyond the right atrium. Hemodialysis catheters were removed significantly earlier after insertion (median 43.08 months) than venous ports (median 78.53 months).

**Table 1:** Patients and their permanent catheters

**Demographic, clinical and catheter data**	**Permcaths**	**Vascular port and other**	**Chi^2^, Mann-Whitney U tests P**	**All catheters**
	**N (%) median** **(Q1; Q3)**	**N (%) median** **(Q1; Q3)**		**N (%) median** **(Q1; Q3)**
Number of patients	70 (100.0)	41 (100.0)		111 (100.0)
Females	30 (42.86)	29 (70.73)	0.008	59 (53.15)
Males	40 (57.14)	12 (29.27)	0.008	52 (46.85)
EF < 40%	14 (20.00)	1 (2.44)	0.020	15 (13.51)
Congestive heart failure	23 (32.86)	3 (7.32)	0.005	26 (23.42)
Diabetes mellitus (any)	22 (31.43)	4 (9.76)	0.018	26 (23.42)
Ischemic heart disease, vascular disease	30 (42.86)	4 (9.76)	0.001	34 (30.63)
Permanent atrial fibrillation	10 (14.29)	1 (2.44)	0.092	11 (9.91)
Thrombus on the catheter	6 (8.57)	3 (7.32)	0.899	9 (8.11)
Anticoagulation	27 (38.57)	5 (12.20)	0.006	32 (28.83)
Removed catheter location (chest side)				
Right	45 (64.29)	23 (56.10)	0.514	68 (61.26)
Left	23 (32.86)	16 (39.02)	0.652	39 (35.14)
Both	2 (2.86)	2 (4.88)	0.981	4 (3.60)
Indications for catheter removal				
Localized (venous entry) infection	2 (2.86)	1 (2.44)	0.635	3 (2.70)
Systemic infection or endocarditis	23 (32.86)	7 (17.07)	0.113	30 (27.03)
Systemic - vegetation (subgroup of endocarditis)	12 (17.14)	6 (14.63)	0.937	18 (16.22)
Mechanical catheter damage	0 (0.00)	3 (7.32)	0.091	3 (2.70)
Catheter malfunction	43 (61.43)	20 (48.78)	0.272	63 (56.76)
No longer needed	2 (2.86)	10 (24.39)	0.001	12 (10.81)
All indications	70 (100.0)	41 (100.0)		111 (100.0)
Underlying disease				
Renal failure	70 (100.0)	0 (0.00)	< 0.001	70 (63.06)
Neoplastic disease	0 (0.00)	34 (82.93)	< 0.001	34 (30.63)
Parenteral nutrition or another permanent catheter	0 (0.00)	7 (17.07)	0.002	7 (6.31)
All underlying diseases	70 (100.0)	41 (100.0)		111 (100.0)
Presence of CIED system				
Pacemaker	9 (12.86)	1 (2.44)	0.132	10 (9.01)
ICD	5 (7.14)	0 (0.00)	0.202	5 (4.50)
CRT-D	3 (4.29)	0 (0.00)	0.461	3 (2.70)
Micra	1 (1.43)	0 (0.00)	0.786	1 (0.90)
Lack of CIED	52 (74.29)	40 (97.56)	0.004	92 (82.88)
Intracardiac lead location				
Left side of the chest	12 (17.14)	0 (0.00)	0.013	12 (10,81)
Right side of the chest	2 (2.86)	0 (0.00)	0.724	2 (1,80)
CIED system duration [months]	72.93 (35.80-94.07)	165.10 (one case)	NC	74.52 (35.80; 108.6)
Catheter location (chest side)				
Left side of the chest	23 (32.86)	16 (39.02)	0.652	39 (35.14)
Right side of chest	45 (64.29)	23 (56.10)	0.514	68 (61.26)
Both sides	2 (2.86)	2 (4.88)	0.981	4 (3.60)
All	70 (100.0)	41 (100.0)		111 (100.0)
Catheter dwell time [months]	43.08 (22.43; 6.37)	78.53 (28.7;123.8)	0.013	54.07 (28.20; 82.80)
Location of catheter tip				
Brachiocephalic trunk	8 (11.43)	6 (14.63)	0.846	14 (12.61)
Vena cava superior	22 (31.43)	16 (39.02)	0.544	38 (34.23)
High right atrium	2 (2.86)	1 (2.44)	0.635	3 (2.70)
Mid right atrium	32 (45.71)	10 (24.39)	0.042	42 (37.84)
Low right atrium	2 (2.86)	1 (2.44)	0.635	3 (2.70)
Tricuspid valve	0 (0.00)	2 (4.88)	0.260	2 (1.80)
Vena cava inferior	4 (5.71)	0 (0.00)	0.302	4 (3.60)
Pulmonary artery	0 (0.00)	5 (12.20)	0.012	5 (4.50)
All	70 (100.0)	41 (100.0)		111 (100.0)

Abbreviations: EF, ejection fraction, CIED, cardiovascular implantable electronic devices, ICD, implantable cardioverter-defibrillator, CRT-D, implantable cardiac defibrillator with ventricular resynchronization.

**Figure 4:**
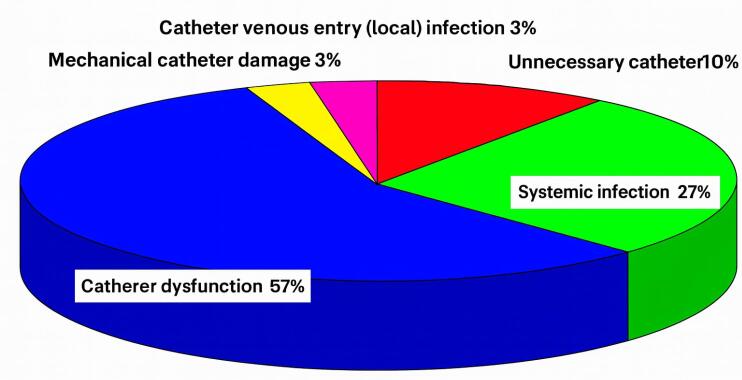
Indications for adherent catheter removal

 Indications (percentage) for transvenous catheter extraction are displayed on the graph (Figure 4).

Table 2 compares transvenous removal of adherent hemodialysis catheters and venous ports. During the extraction of both types of venous devices mechanical dilatation was necessary in more than half of cases. In half of patients with their hemodialysis catheter removed a new device was needed. In contrast, almost all venous ports were removed without the need for a new one as they were no longer needed. Among patients with CIEDs in rare cases a hemodialysis catheter and an intracardiac lead were removed at the same time. The main indication for this double procedure was systemic infection. The efficacy of TCE procedures was 100%, and the rate of major complications (one event per all procedures) had no statistical power.

**Table 2:** Transvenous catheter extraction (TCE)

**Transvenous catheter extraction or removal procedure**	**Permcaths**	**Vascular ports and other permanent catheters**	**Chi^2^, Mann-Whitney U tests P**	**All catheters**
	N (%)	N (%)		N (%)
Number of patients	70 (100.0)	41(100.0)		111 (100.0)
Methodology for catheter removal				
Simple traction without keeping approach	29 (41.43)	21(51.22)	0.422	50 (45.05)
Simple traction with keeping venous approach	6 (8.57)	2 (4.88)	0.729	8 (7.21)
Mechanical dilatation without keeping approach	26 (37.14)	18 (43.90)	0.616	44 (39.64)
Mechanical dilatation with keeping venous approach	8 (11.43)	0 (0.00)	0.062	8 (7.21)
Two-step procedure	1 (1.43)	0 (0.00)	0.786	1 (0.90)
All procedures	70 (100.0)	41(100.0)		111 (100.0)
Type of procedure				
Only catheter removal / extraction	49 (70.00)	40 (97.56)	0.001	89 (80.18)
Catheter replacement using the same approach	12 (17.14)	0 (0.00)	0.013	12 (10.81)
Catheter replacement using alternative approach	8 (11.43)	1 (2.44)	0.189	9 (8.11)
Two-step procedure	1 (1.43)	0 (0.00)	0.786	1 (0.90)
All procedures	70 (100.0)	41 (100.0)		111 (100.0)
Hybrid procedures				
Simultaneous or subsequent CIED system removal	7 (10.00)	0 (0.00)	0.092	7 (6.31)
Subsequent venoplasty with stenting	8 (11.43)	4 (9.76)	0.966	12 (10.81)
TCE outcomes				
Successful catheter removal	70 (100.0)	41 (100.0)		111 (100.0)
Unsuccessful catheter dilatation	0 (0.00)	1 (2.44)	0.786	1 (0.00)
Major complications	0 (0.00)	1 (2.44)	0.786	0 (0.00)
Procedure-related death	0 (0.00)	0 (0.00)	NC	0 (0.00)
Indication-related death	1 (1.43)	1 (2.44)	0.724	2 (1.80)
A new catheter after TCE				
Not implanted	16 (22.86)	39 (95.12)	< 0.001	55 (49.55)
Temporary / acute	21 (30.00)	1 (2.44)	0.001	22 (19.82)
Permanent	33 (47.14)	1 (2.44)	< 0.001	34 (30.63)
All procedures	70 (100.0)	41 (100.0)		111 (100.0)

Abbreviations: CIED, cardiovascular implantable electronic devices, TCE, transvenous catheter extraction

 The analysis showed that patient-dependent or device-dependent (catheter/venous port) factors did not affect significantly the need for mechanical dilatation during TCE (Table 3).

**Table 3:** Identification of factors predicting the need for mechanical dilation

**Potential factors predicting the need for mechanical dilatation**	**Mechanical dilatation**	**Simple traction**	**Chi^2^, Mann-Whitney U tests P**	**All catheters**
Number of procedures	53	58		111
Potential factors predicting the need for mechanical dilation	N (%) median (Q1; Q3)	N (%) median (Q1; Q3)		N (%) median (Q1; Q3)
Patient age during TCE [years]	59.00 (42.00-69.00)	58.50 (45.00-72.00)	0.690	59.00 (44.00; 70.00)
Low EF ( < 40%)	8 (15.09)	7 (12.07)	0.851	15 (13.51)
Infection (any)	13 (24.53)	20 (34.48)	0.348	33 (29.73)
Catheter dysfunction / damage	32 (60.38)	34 (58.62)	0.996	66 (59.46)
Unnecessary catheter or restoration of venous access	8 (15.09)	4 (6.90)	0.279	12 (10.81)
Presence of endocardial leads	9 (16.98)	8 (13.79)	0.840	17 (15.32)
Presence of renal failure	35 (66.04)	35 (60.34)	0.672	70 (63.06)
Chemotherapy	15 (28.30)	19 (32.76)	0.762	34 (30.63)
Catheter dwell time [months]	42.77 (21.60-72.37)	60.80 (37.83-92.53)	0.157	54.07 (28.20; 82.80)
Abnormal catheter tip position (outside of RA)	28 (52.83)	35 (60.34)	0.544	63 (56.76)
Catheter insertion on the left side of the chest	21 (39.62)	18 (31.03)	0.455	39 (35.14)

Abbreviations: TCE, transvenous catheter extraction, EF, ejection fraction, RA, right atrium.

Table 4 compares potential risk factors for infection in patients with hemodialysis catheters and venous ports. Patients with catheter-related infections were less likely to have an arteriovenous fistula and intracardiac leads.

**Table 4:** Identification of risk factors for catheter infection

**Potential risk factors for catheter infection**	**Infection (any)**	**Non-infectious removal (any)**	**Chi^2^, Mann-Whitney U tests P**	**All catheters**
Number of procedures	33	78		111
Potential risk factors for catheter-related infection	N (%) median (Q1; Q3)	N (%) median (Q1; Q3)		N (%) median (Q1; Q3)
Patient age during TCE [years]	57 (43.00- 72.00)	59.00 (44.00-68.00)	0.874	59.00 44.00; 70.00)
Low EF	3 (9.09)	12 (15.38)	0.560	15 (13.51)
Diabetes mellitus	9 (27.27)	17 (21.79)	0.706	26 (23.42)
Permanent AF	2 (6.06)	9 (11.54)	0.592	11 (9.91)
Presence of endocardial leads	9 (27.27)	8 (10.26)	0.047	17 (15.32)
Presence of AV fistula	13 (39.39)	20 (25.64)	0.222	33 (29.73)
Presence of renal failure	25 (75.76)	45 (57.69)	0.006	70 (63.06)
Chemotherapy	5 (15.15)	29 (37.18)	0.034	34 (30.63)
Catheter dwell time [months]	51.28 (20.25; 68.74)	56.30 (30.27; 90.60)	0.528	54.07 (28.20; 82.80)
Abnormal catheter tip position (outside of RA)	14 (42.42)	49 (62.82)	0.076	63 (56.76)
Catheter insertion on the left side of the chest	8 (24.24)	31 (39.74)	< 0.001	39 (35.14)

Abbreviations: TCE, transvenous catheter extraction, EF, ejection fraction, AF, atrial fibrillation, AV fistula, arteriovenous fistula, RA, right atrium.

 The right-sided location of hemodialysis catheters was associated with more frequent device infections. Placement on the left side of the chest was related to more frequent mechanical dilatation during TCE, symptomatic venous obstruction and improper position of the catheter tips. However, the data did not achieve statistical significance (Table 5).

**Table 5:** Differences between right- and left-sided catheter location

**Catheter insertion route (chest side)**	**Right approach**	**Left (or both) approach**	**Chi^2^, Mann-Whitney U tests P**	**All patients / catheters**
Potential factors predicting the need for catheter removal and level of procedure complexity	N (%)	N (%)		N (%)
Number of patients	68 (100.0)	43 (100.0)		111 (100.0)
Indications for TCE				
Infection (any)	24 (35.29)	9 (20.93)	0.162	33 (29.73)
Mechanical catheter damage	1 (1.47)	2 (4.65)	0.685	3 (2.70)
Catheter malfunction	38 (55.88)	25 (58.14)	0.970	63 (56.76)
Unnecessary catheter	5 (7.35)	7 (16.28)	0.245	12 (10.81)
All	68 (100.0)	43 (100.0)		111 (100.0)
Methods of TCE				
Removal without dilatation	38 (55.88)	20 (46.51)	0.443	58 (52.25)
Mechanical dilatation necessary	29 (42.65)	23 (53.49)	0.358	52 (46.85)
Two-step procedure	1 (1.47)	0 (0.00)	0.816	1 (0.90)
All TCEs	68 (100.0)	43 (100.0)		111 (100.0)
Symptomatic venous stenosis / occlusion				
Present	4 (5.88)	8 (18.60)	0.074	12 (10.81)
Absent	64 (94.12)	35 (81.40)	0.074	99 (89.19)
All	68 (100.0)	43 (100.0)		111 (100.0)
Tip location				
Improper catheter tip position (ALL BUT RA)	34 (50.00)	29 (67.44)	0.107	63 (56.76)
Proper tip position (inside RA)	34 (50.00)	14 (32.56)	0.107	48 (43.24)
All TCEs	68 (100.0)	43 (100.0)		111 (100.0)

Abbreviations: TCE, transvenous catheter extraction, RA, right atrium.

Table 6 summarizes the data on the incidence of arteriovenous fistulas among patients undergoing TCE. Less than half of the patients had an arteriovenous fistula, most of which were inactive. In all patients with an arteriovenous fistula and CIED, the leads were placed on the side of the fistula (both active and inactive).

**Table 6:** Presence of an arteriovenous (AV) fistula for hemodialysis

**Type of intravascular catheter**	**Permcaths**	**Vascular ports and other permanent catheters**	**Chi^2^ test P**	**All catheters**
Presence of AV fistula for hemodialysis	N (%)	N (%)		N (%)
Lack of AV fistula	37 (52.86)	41 (100.0)	< 0.001	78 (70.27)
Left side functional	7 (10.00)	0 (0.00)	0.092	7 (6.31)
Right side functional	6 (8.57)	0 (0.00)	0.136	6 (5.41)
Right side out of order	2 (2.86)	0 (0.00)	0.724	2 (1.80)
Left side out of order	8 (11.43)	0 (0.00)	0.062	8 (7.21)
Both sides out of order	10 (14.29)	0 (0.00)	0.028	10 (9.01)
All	70 (100.0)	41 (100.0)		111 (100.0)
Fistula on the lead placement side	8 (11.42)	0 (0.00)	0.062	8 (7.21)
Fistula on the side opposite to the leads	0 (0.00)	0 (0.00)	NC	0 (0.00)

Abbreviations: AV fistula, arteriovenous fistula.

## Discussion

 Patients undergoing transvenous removal of adherent hemodialysis catheters and venous ports frequently have comorbidities which may modify the course of the underlying disease,^4,9^ and increase the procedure-related risk.

 The most common indication for removal of hemodialysis catheters and venous ports is catheter malfunction. Malfunction is defined as complete obstruction (catheters are unable to perform hemodialysis and venous ports fail to administer chemotherapy and parenteral nutrition) or significant limitation of its function (too much resistance on blood withdrawal) due to fibrous sheath at the tips or adherence to heart structures.^4,9-12^ The second is infection, both systemic and localized. Because of the presence of artificial material in the venous system or in the right atrium it is not surprising that infective endocarditis on the right side of the heart is more common than in the general population.^27,28^ The detection of a vegetation attached to the catheter on imaging tests is one of major Duke criteria for the diagnosis of infective endocarditis.^26^ The indications listed for the removal of dialysis catheters or vascular ports are consistent with the indications described in the available literature. The most common imaging test in our registry was transthoracic echocardiography (TTE), and if possible, followed by transesophageal echocardiography (TEE).^26,29-30^ Much less common were other imaging tests such as computerized tomography (CT) of the heart or magnetic resonance imaging (MRI). Recommended by the European Society of Cardiology radioisotope studies such as positron emission tomography (PET) or single photon emission computed tomography (SPECT) were rare.^26,29-30^ Unfortunately, these tests are much less available and rarely performed in the diagnostic process in patients with catheter-related infective endocarditis. Radioisotope studies additonally provide valuable information in patients with artificial materials in the heart (for instance patients with artificial heart valves) and in patients with the presence of endocardial leads. Apart from TTE and TEE, additional imaging studies increase the likelihood of differentiating between vegetation and thrombus. Therefore, the detection of vegetation (or suspected vegetation as a matter of fact) based on TTE and TEE, without meeting other Duke criteria, cannot provide grounds for diagnosis of catheter-related infective endocarditis.^8-9,29-33^ The third indication for removal is no longer needed venous port, most frequently associated with termination of chemotherapy, and creation of an arteriovenous fistula eliminating the need for a hemodialysis catheter.

 When analyzing potential risk factors for catheter infection or catheter-related systemic infection the following conventional factors were taken into account, namely diabetes mellitus, heart failure with reduced left ventricular ejection fraction, renal failure or the presence of an additional artificial material in the venous system and the heart – in this case the presence of an endocardial lead.^4,9^ However, we did not demonstrate that catheter-related infections were statistically more frequent in these patients. It was demonstrated that placement of a catheter on the left side of the chest (most frequently the catheter inserted via the internal jugular vein or left axillary/subclavian vein) was associated with lower risk of catheter infection.

 New venous ports were also significantly less often implanted after removal as they were no longer needed in contrast to hemodialysis catheters. Generally, an unnecessary or infected catheter is removed whereas malfunctioning non-infected catheter is exchanged for a new one via the same venous access site if possible.^4,10-12^

 In our registry of the patients undergoing transvenous catheter removal, mechanical dilatation was indispensable in 48.57% of patients with hemodialysis catheters and in 43.9% of patients with venous ports. In 42.65% of cases catheters removed using mechanical dilatation were placed on the right side and in 53.49% on the left side of the chest. Mechanical dilatation was 100% successful for removal of both hemodialysis catheters and venous ports, one patient with a hemodialysis catheter required two-step procedure for successful removal. In the available medical literature, there are studies concerning the removal of catheters; however, most of them do not describe the removal technique itself or describe the Hong’s technique. Therefore, we cannot compare the results of this study with the available literature, as there is a lack of data on catheter removal using the described method.

 The factors that predicted the use of additional tools such as polypropylene telescoping sheaths during transvenous removal of adherent hemodialysis catheters and venous ports included renal failure, catheter malfunction as an indication for the procedure, and improper position of the catheter tip. Renal failure increases the risk of vessel calcification, smooth muscle cells transform into chondrocytes or osteoblast-like cells, which appears to be the key element in the pathogenesis of the process in the context of passive deposition of calcium and phosphates due to metabolic bone disorders and decreased renal excretion.^34-36^ The same mechanism probably predisposes patients treated with hemodialysis to calcification and catheter adherence to the vessel wall or the heart.^34-36^ Improper position of the catheter tip causing mechanical irritation of the vein wall affects catheter function (shorter time to its malfunction) and favors the development of venous obstruction.^4,9-12^ There was no significant difference between predictors of mechanical dilatation between patients with hemodialysis catheters and with venous ports.

 An urgent indication for transvenous removal of hemodialysis catheters/venous ports is dislodgement of catheter/port tips or their fragments. Most often, the catheter is ruptured due to mechanical damage, the tip is dislodged into the venous system, right atrium or pulmonary artery (four cases). Dislodgement of the entire catheter is markedly less likely, but the size of dislodged fragments may vary. A retained foreign body increases the risk of pulmonary embolism, thrombosis and infection involving the ruptured fragment and surrounding structures. The ruptured fragment is often detected accidentally during diagnostic imaging. The procedure of removing the dislodged catheter or its fragment is similar to the procedure of TCE and TLE (with respect to personnel, venue and patient preparation). The only difference is the technique of grabbing the retained fragment. In this case we create a convenient, additional access to a large vein, most frequently the femoral vein, insert a sheath through which a dedicated “lasso” catheter (loop snare, pigtail catheter and goose neck snare) is advanced. The whole procedure is performed under fluoroscopy control. The retained piece is grabbed with the tightened “lasso” loop and easily removed. Depending on further indications some patients receive a new venous port.^37-41^

 Based on the results of our single center registry and experience of other centers transvenous removal of adherent hemodialysis catheters and venous ports is a safe procedure and can be recommended as first-choice treatment option.^42-48^ Performing TCE we avoid large and traumatic operations, often associated with numerous complications requiring thoracotomy, opening of the superior vena cava or brachiocephalic trunk. We may offer the patient a minimally invasive procedure with a high probability of complete success and reduction of possible complications.^42-48^ In the literature, there are other removal techniques such as endoluminal balloon dilatation of the dialysis catheter.^47-48^ Endoluminal balloon dilatation separates the catheter by breaking the adhesions between the catheter and the vein. In this way, we also gain access to the vein lumen (we create vascular access through placement of a guide wire which may also be used for insertion of a new catheter), removal of adherent and obstructed catheter becomes simpler and safer (Hong’s technique).^47-48^

 In all patients with CIEDs and arteriovenous fistula, the CIED was always on the same side of the chest as the fistula. However, CIED placement on the same side of the chest as the arteriovenous fistula should be avoided because of the increased risk of venous thrombosis and fistula failure.^49^

 Winding up, a larger number of catheter insertions is associated with a larger number of complications. This in turn forces more frequent removals of adherent catheters and venous ports, search for new more effective technical solutions and improvement of procedure organization.

 This study presents the experience of a single center in a relatively small group of patients. This is an observational, retrospective study. A large number of patients did not have echocardiography before the procedure. No laser energy was used during the procedure. Endoluminal balloon dilatation is known to the authors but limited experience does not allow to draw any conclusions.

## Conclusion

1. The most frequent indication for catheter removal is its malfunction, usually due to fibrous sheath on its tips or adherence of the tip to cardiac structures. The second most frequent indication is catheter-related infection.
2. Most of failed and even infected catheters is implanted with improper position of the tip outside of the right atrium.
3. Half of seemingly adherent catheters can be removed with simple traction, but we are not able to predict this before the procedure. Half of catheters requires separation from fibrous sheath using additional dedicated tools.
4. The final result of mechanical dilatation is very good if the procedure is performed with collaboration of operators experienced with transvenous lead extraction.
5. Transvenous removal of adherent hemodialysis catheters and venous ports is safe and effective.

There is a need to expand the existing patient database, as well as to conduct further studies in order to enhance our knowledge of the factors that could allow us to predict the necessity of mechanical dilatation during the removal of dialysis catheters or vascular ports. Moreover, additional research is needed to identify the factors that predispose to dysfunction or infection of the implant.

## Competing Interests

 Authors declare no conflict of interest.

## Ethical Approval

 The retrospective analysis of patient documentation was performed in accordance with the *Declaration of Helsinki* principles and after approval of the Bioethics Committee in Lublin no. 288/2018/KB/VII. Before the procedure each patient signed the informed consent to process his/her study results and agreed to be contacted on the phone to provide health update.
